# Angiomyolipoma With Epithelial Cysts: A Rare but Distinct Variant of Angiomyolipoma

**DOI:** 10.7759/cureus.51824

**Published:** 2024-01-07

**Authors:** Vijai R, Pritam Sharma, Parth A Patel, Pratik Patil

**Affiliations:** 1 Urology, A.J. Institute of Medical Sciences, Mangalore, IND

**Keywords:** pancytokeratin, infertility, renal lesion, epithelial cyst, angiomyipoma

## Abstract

Renal angiomyolipomas, common benign tumors, can exhibit slow growth in sporadic cases or have aggressive tendencies when linked to genetic conditions like tuberous sclerosis. This case report focuses on the exceptionally rare angiomyolipoma with epithelial cysts (AMLEC) variant, particularly challenging to diagnose due to its scarcity. Describing a 41-year-old woman's case, initially suspected to be renal cell carcinoma during an infertility evaluation, subsequent partial nephrectomy revealed a tumor comprising smooth muscle, blood vessels, and fat, with cystic regions featuring cuboidal linings and a layer devoid of abnormal cell activity. Immunohistochemistry confirmed specific markers within different tumor components, highlighting the diagnostic complexities of AMLEC and emphasizing the crucial role of histopathological examinations in accurate characterizations.

## Introduction

Renal angiomyolipomas represent the most common category of benign renal tumors detected incidentally [1]. These solid, highly vascular growths predominantly consist of smooth muscle cells, blood vessels, and varying proportions of adipocytes, with cystic components being a rare occurrence [2].

While approximately 80% of renal angiomyolipomas occur sporadically, roughly 20% of cases have a hereditary connection to conditions like tuberous sclerosis and pulmonary lymphangioleiomyomatosis. Sporadic cases tend to be more benign, featuring smaller, stable, unilateral lesions with steady growth patterns, in contrast to hereditary variants. Hereditary angiomyolipomas often present as larger, bilateral lesions with aggressive growth tendencies and an earlier age of onset. Those associated with tuberous sclerosis frequently involve genetic mutations in the TSC1 and TSC2 genes, known to impact the mTOR receptor [2,3].

The literature describes various morphologic variations of angiomyolipomas, including epithelioid, oncocytic, fat-dominant, smooth muscle-dominant, and those with epithelial cysts or sclerosing characteristics [4-6]. Among these variants, angiomyolipoma with epithelial cysts (AMLEC), also known as cystic angiomyolipoma, is exceptionally rare [7]. There is limited documentation in case series and reports, underscoring its unusual nature [8]. While AMLEC primarily affects females and generally manifests as benign, well-defined, and slowly growing lesions, sporadic instances hint at possible associations with tuberous sclerosis complex and co-occurrence with solid-cystic renal cell carcinoma.

Despite the essential role of radiological examinations through ultrasound and contrast-enhanced computed tomography (CECT) in diagnosis, AMLEC often resembles more common radiological entities, such as cystic renal cell carcinoma (RCC), cystic nephroma (CN), or mixed epithelial and stromal tumor (MEST), due to its rarity [9]. Consequently, histopathological examination remains the gold standard for reaching a definitive diagnosis, with immunohistochemistry and molecular markers playing a pivotal role in characterizing these lesions [10].

In this case report, we explore the intriguing landscape of renal angiomyolipomas, shedding light on the fascinating interplay between sporadic and hereditary occurrences, as well as the enigmatic AMLEC variant. We emphasize the diagnostic challenges posed by the rarity of AMLEC and the pivotal role of histopathological and immunohistochemical examinations in unraveling its unique character.

## Case presentation

**Figure 1:**
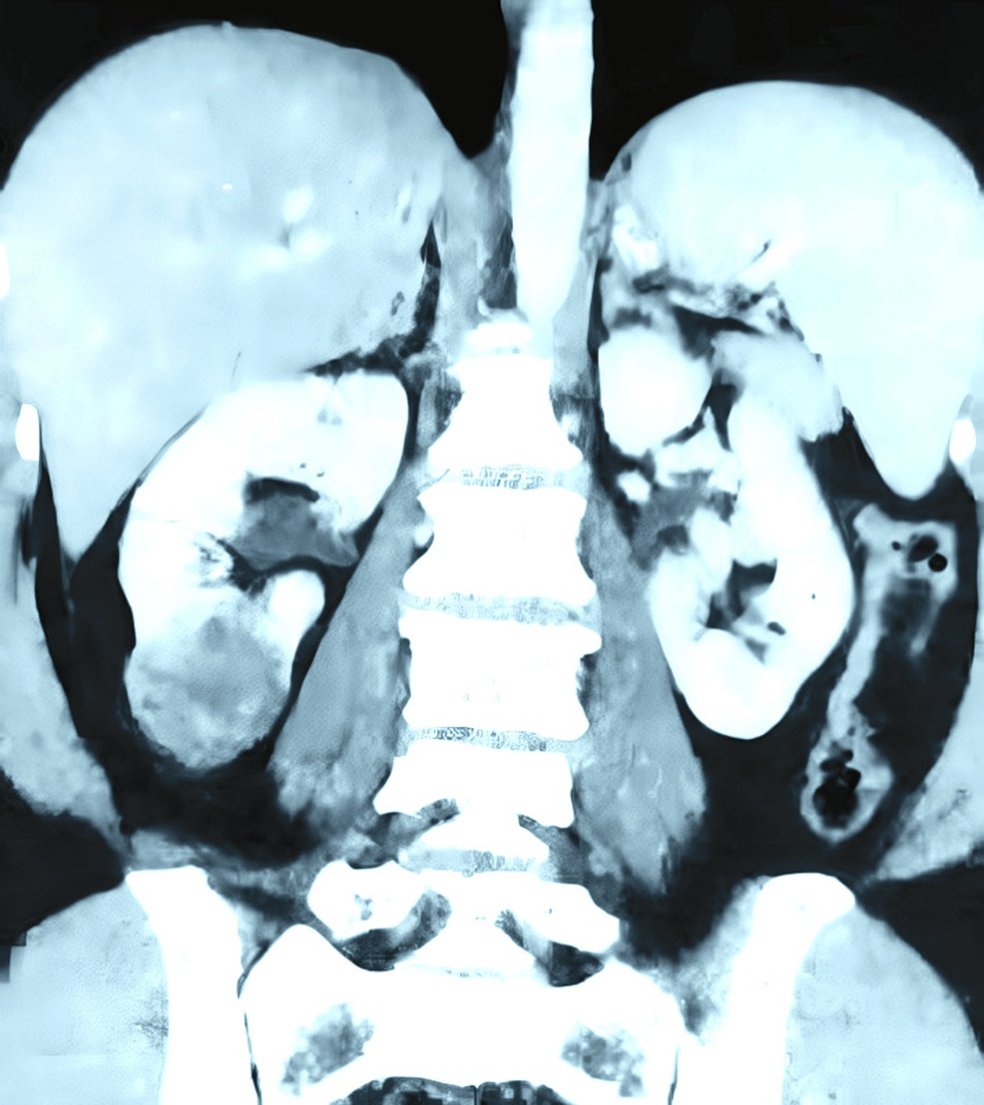
A well-defined heterogeneously enhancing solid cystic mass lesion measuring approximately (3.8 x 3.6 x3.4 cm) involving the lower pole of the right kidney There was no evidence of extension of the lesion into the pararenal space and the right renal artery and vein appeared normal radiologically; this was provisionally diagnosed as renal cell carcinoma (RCC).

**Figure 2:**
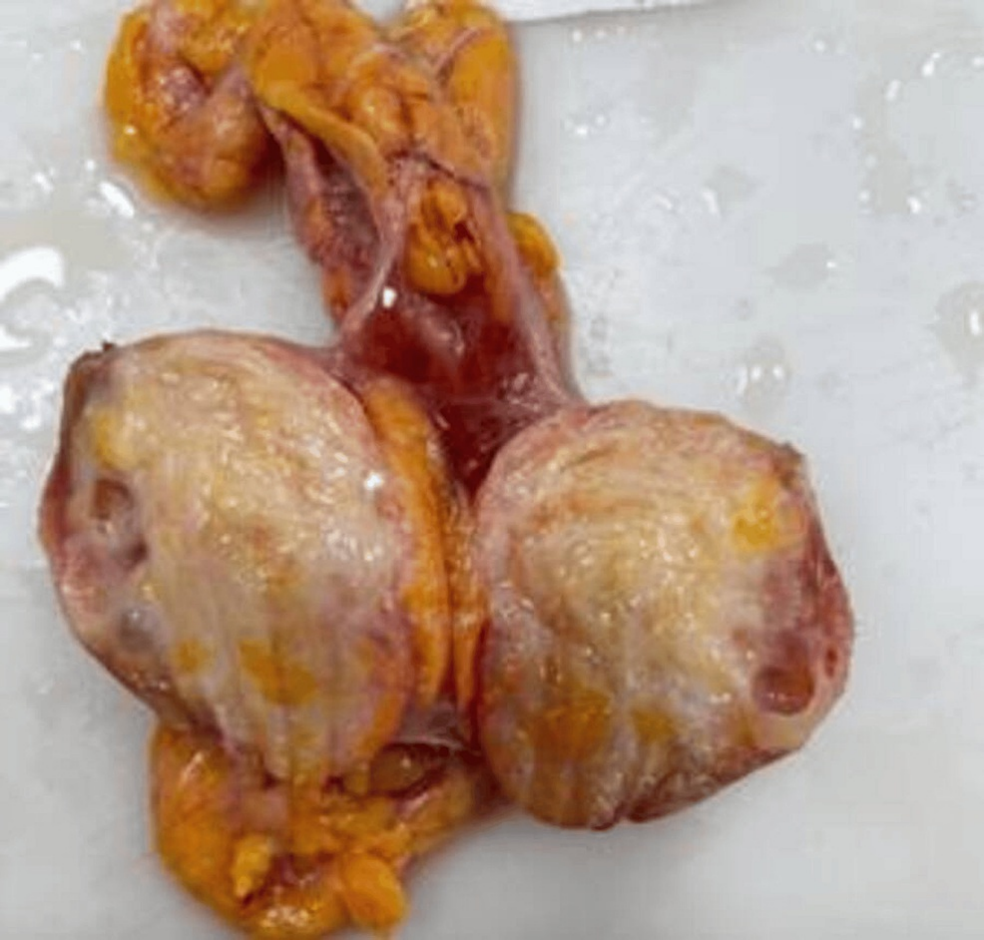
The kidney was normal, but the cut surface showed a solid mass with focal cystic areas (3 x 2 cm) occupying the lower pole of the kidney The solid area was firm, glistening white to focally yellowish.

**Figure 3:**
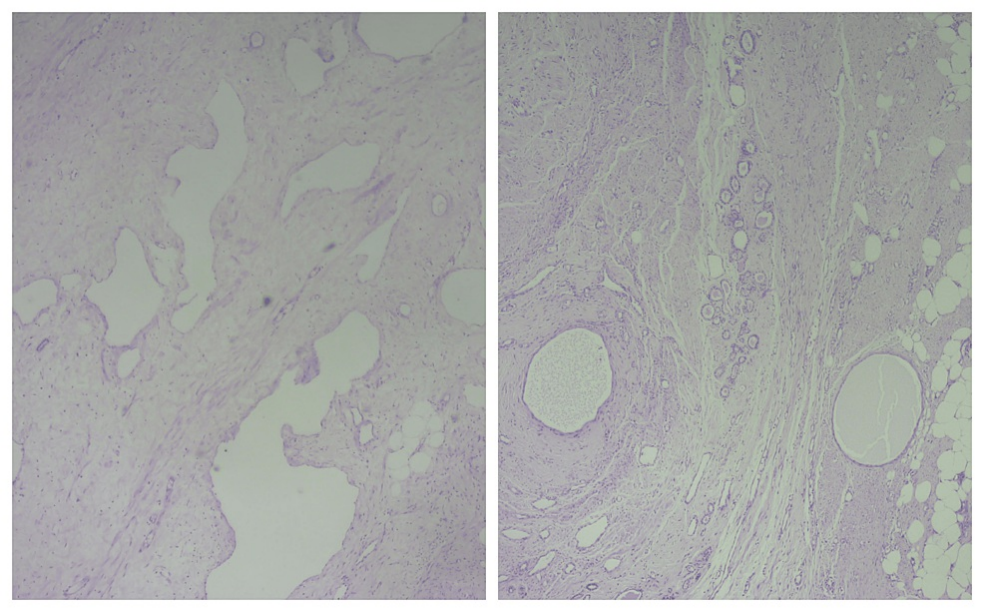
Histopathological examination (HPE) revealed a well-circumscribed tumor with an intimate mix of smooth muscle, disorganized blood vessels, and adipose tissues

**Figure 4:**
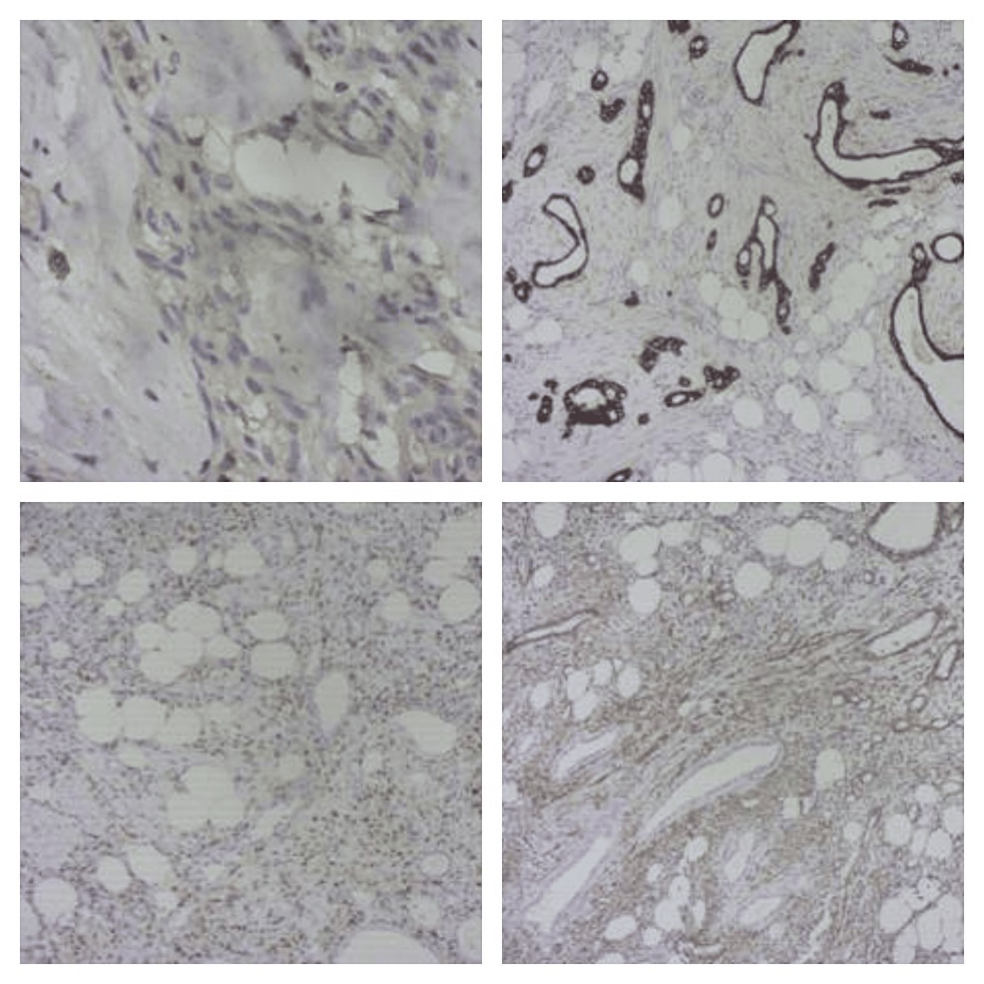
On immunohistochemistry (IHC), the epithelial lining of cystic spaces showed positivity for pancytokeratin (CK), estrogen-receptor alpha (ER) seen in stromal cells, smooth muscle actin (SMA) strongly positive in the stromal component and blood vessels; patchy weakly positive HMB45 seen in the cambium layer

A 41-year-old female patient sought medical attention due to an incidental finding of a renal lesion while undergoing infertility evaluation. She reported no history of hematuria, and a physical examination revealed a soft abdomen with no palpable mass. Subsequently, a contrast-enhanced computed tomography scan (CECT) revealed a well-defined, heterogeneously enhancing, solid cystic mass in the lower pole of her right kidney, measuring approximately 3.8 x 3.6 x 3.4 cm as shown in Figure 1. Radiologically, it was provisionally diagnosed as renal cell carcinoma (RCC), despite normal findings in the urinalysis, sterile urine culture, and within-normal-limits routine laboratory investigations. The patient underwent a right open partial nephrectomy, with a ureteric catheter placed via cystoscopy before the procedure. A gross examination of the kidney showed a normal appearance with a solid mass, including focal cystic areas measuring 3 x 2 cm, primarily occupying the lower pole as shown in Figure 2. Histopathological examination (HPE) revealed a well-circumscribed tumor with an intimate mix of smooth muscle, disorganized blood vessels, and adipose tissues, where smooth muscle predominated. The tumor displayed a mixed solid and cystic architecture, with cuboidal epithelial linings in the cysts and a compact cellular layer underneath as shown in Figure 3. Notably, there was an absence of nuclear atypia and mitotic activity. Immunohistochemistry (IHC) demonstrated positive staining for pancytokeratin (CK) in the epithelial lining of the cystic spaces, estrogen-receptor alpha (ER) in stromal cells, and strong smooth muscle actin (SMA) positivity in the stromal component and blood vessels, along with weakly positive HMB45 in the cambium layer as shown in Figure 4. A one-month follow-up post-surgery indicates the patient's satisfactory recovery with no complaints.

## Discussion

Angiomyolipoma with epithelial cysts (AMLEC) stands as a rare variant of AML, with limited documented cases in the literature. Typically, AMLECs are observed in middle-aged individuals, displaying a slight female predilection. Much like traditional AML, they often remain asymptomatic and are incidentally discovered during the assessment of other abdominal pathologies [4]. The pathogenesis of AMLEC remains a subject of debate, with the predominant theory being the entrapment of native epithelial cells leading to secondary cystic dilatation while another theory suggests epithelial differentiation into cystic elements [7].

Radiologically and grossly, AMLECs present as either unilocular or multilocular cystic lesions, frequently containing serous fluid and, at times, blood [7]. These cystic elements coexist with a sparse amount of fat and solid components, occasionally manifesting as mural nodules [7,11]. This variable composition, coupled with its rarity, often complicates diagnosis, leading to misclassification as renal cell carcinoma (RCC) or cystic nephroma.

In the case we presented, our patient was asymptomatic, and a CECT-abdomen pelvic examination conducted to explore potential causes of primary infertility revealed a well-defined, heterogeneously enhancing solid-cystic lesion in the lower pole of the right kidney. Radiologically and macroscopically, the provisional diagnosis pointed to renal cell carcinoma. However, histopathological examination (HPE) and immunohistochemistry (IHC) emerged as the gold standard for the definitive diagnosis. The HPE showed well-differentiated cells with an amalgamation of smooth muscle cells, blood vessels, adipose tissues, and multicystic elements lined with cuboidal epithelium. There was an absence of nuclear atypia or mitosis in any of the cellular components examined.

IHC staining indicated positive results for pancytokeratin (CK) in the epithelial lining of cystic spaces, estrogen receptor alpha (ER) in stromal cells, smooth muscle actin (SMA) in the stromal component and blood vessels, and weakly positive HMB45 in the cambium layer. Several conditions present with a morphological resemblance to AMLEC, including renal cell carcinoma (RCC), cystic nephroma, and mixed epithelial and stromal tumor (MEST). However, differentiation through HPE can be performed with a reasonable degree of confidence. For instance, cystic RCC exhibits nuclear atypia, cystic nephroma displays dysplastic thick-walled vessels with cellular ovarian-type stroma, and cystic partially differentiated nephroblastoma (CPDN) features undifferentiated blastemal components that stain positively for WT1 (Wilms tumor 1) [12].

In an article published by Woo Cheal Cho et al., a case report on a 46-year-old woman with tuberous sclerosis complex (TSC) who was diagnosed with eosinophilic solid and cystic renal cell carcinoma (ESCRCC) and two synchronous angiomyolipomas, one of which was a rare cystic variant of AML called angiomyolipoma with epithelial cysts (AMLEC). The authors also identified additional copy number alterations in ESCRCC via molecular karyotyping [13]. The report also describes a unique histologic feature of TSC-associated ESCRCC, which has not been previously described in detail. The feature involves the formation of semicircular multinucleated neoplastic giant cells engulfing an additional intact neoplastic cell, simulating emperipolesis.

Also in another case, X Zhang et al. presented findings on four female patients, aged 19 to 52 years (mean 34.5 years), with renal tumors. Three cases were incidentally discovered during physical examinations, with medical histories ranging from one to six years. Preoperative imaging revealed Bosniak classifications of grade Ⅲ in three cases and grade Ⅳ in one case. Tumor diameters ranged from 2.5 to 9.0 cm (average 5.0 cm). Histologically, all tumors exhibited three components: simple epithelial cysts with cuboidal/low-columnar to occasional hobnail cells; a thin subepithelial "cambium-like" layer of mullerian-like spindle cell stromas with chronic inflammation; an outer layer of thick, long-fascicles of smooth muscle-like stromas surrounded by dysplastic, tortuous blood vessels, often with a prominent lymphatic channel network. None of the tumors contained fat [14]. Immunohistochemically, cyst-lining epithelial cells expressed PAX8 and CK7, while the "cambium-like" stroma expressed melanocytic and Mullerian markers but were negative for smooth-muscle markers. The outermost smooth muscle-like stromas strongly expressed smooth-muscle markers and were only sporadically positive for melanocytic and Mullerian markers. Follow-up information for three cases showed no evidence of tumor recurrence or metastasis at 3, 5, and 66 months, respectively.

Regarding the management of AMLEC, no standardized protocols or treatments exist due to the benign nature of the lesions. Surgical excision, as part of partial or radical nephrectomy, remains the mainstay for managing large or symptomatic AMLECs. Surgical intervention may also be planned when the lesion's morphology resembles renal cell carcinoma [2]. While renal biopsy theoretically aids in histopathological diagnosis, it is often avoided due to associated complications, such as tumor seeding in RCC or excessive bleeding in AML cases. In the case presented, the lesion was managed similarly to RCC, and an open partial nephrectomy was performed before HPE and IHC examination. With a benign nature, patients have generally demonstrated positive postoperative outcomes and a favorable prognosis following surgical excision [3].

In conclusion, our case highlights the diagnostic challenges and importance of histopathological and immunohistochemical evaluations in confirming AMLEC, a rare renal tumor. Surgical management, often resembling RCC intervention, yields favorable postoperative outcomes due to the benign nature of these lesions, emphasizing the significance of accurate diagnosis and tailored treatment.

## Conclusions

This exploration of renal angiomyolipomas, specifically the rare AMLEC variant, underscores the intricate diagnostic landscape these lesions present. The rarity of AMLEC, often resembling more common renal tumors, necessitates a thorough histopathological and immunohistochemical evaluation to arrive at an accurate diagnosis. The case we presented demonstrates the importance of distinguishing AMLEC from other entities, such as RCC or cystic nephroma, guiding appropriate treatment decisions. Surgical excision remains the primary approach, mirroring RCC management, with the assurance of favorable postoperative outcomes due to the benign nature of AMLEC. This study illuminates the significance of accurate diagnosis and tailored management for rare renal lesions like AMLEC, emphasizing the role of histopathological and immunohistochemical techniques in reaching definitive conclusions.
